# Birth data accessibility via primary care health records to classify health status in a multi-ethnic population of children: an observational study

**DOI:** 10.1038/npjpcrm.2014.112

**Published:** 2015-01-22

**Authors:** Rachel Bonner, Vassiliki Bountziouka, Janet Stocks, Seeromanie Harding, Angela Wade, Chris Griffiths, David Sears, Helen Fothergill, Hannah Slevin, Sooky Lum

**Affiliations:** 1 Respiratory, Critical Care & Anaesthesia Section (Portex Unit), UCL, Institute of Child Health, London, UK; 2 MRC/CSO, Social and Public Health Sciences Unit, University of Glasgow, 4 Lilybank Gardens, Glasgow, UK; 3 Clinical Epidemiology, Nutrition and Biostatistics Section, UCL, Institute of Child Health, London, UK; 4 Centre for Primary Care and Public Health, Blizard Institute, Queen Mary University of London, London, UK; 5 Lung Function Unit, Royal Brompton Hospital, London, UK; 6 Torbay Hospital, South Devon NHS Trust, Torquay, Devon, UK; 7 Faculty of Medicine, University of Southampton, Southampton, UK

## Abstract

**Background:**

Access to reliable birth data (birthweight (BW) and gestational age (GA)) is essential for the identification of individuals who are at subsequent health risk.

**Aims:**

This study aimed to explore the feasibility of retrospectively collecting birth data for schoolchildren from parental questionnaires (PQ) and general practitioners (GPs) in primary care clinics, in inner city neighbourhoods with high density of ethnic minority and disadvantaged populations.

**Methods:**

Attempts were made to obtain birth data from parents and GPs for 2,171 London primary schoolchildren (34% White, 29% Black African origin, 25% South Asians, 12% Other) as part of a larger study of respiratory health.

**Results:**

Information on BW and/or GA were obtained from parents for 2,052 (95%) children. Almost all parents (2,045) gave consent to access their children’s health records held by GPs. On the basis of parental information, GPs of 1,785 children were successfully contacted, and GPs of 1,202 children responded. Birth data were retrieved for only 482 children (22% of 2,052). Missing birth data from GPs were associated with non-white ethnicity, non-UK born, English not the dominant language at home or socioeconomic disadvantage. Paired data were available in 376 children for BW and in 407 children for GA. No significant difference in BW or GA was observed between PQ and GP data, with <5% difference between sources regardless of normal or low birth weight, or term or preterm status.

**Conclusions:**

Parental recall of birth data for primary schoolchildren yields high quality and rapid return of data, and it should be considered as a viable alternative in which there is limited access to birth records. It provides the potential to include children with an increased risk of health problems within epidemiological studies.

## Introduction

Despite increasing evidence that pre-natal and early post-natal insults to the developing lung may affect later respiratory health,^1–4^ remarkably little emphasis has been placed on the need for rapid access to reliable birth data such as birth weight (BW) and gestational age (GA).^5^ Without access to such data, it is difficult to identify individuals who are at subsequent risk, to design intervention studies to prevent or minimise the impact of such insults, or to select a population free of such risks when designing epidemiological studies. Population-based studies of lung function in children often exclude those born preterm (i.e., GA<37 weeks) or those with a low birth weight (LBW, BW<2.5 kg), because of the known long-term influence on lung growth.^6,7^ The most accurate means of obtaining BW and GA data should be via the child’s medical record.^8^ The administrative process to obtain these data from hospitals or primary care centres can, however, be lengthy and complicated. The collection of birth data for many community-based longitudinal epidemiologic studies in the UK is via parental recall.^9,10^ Evidence regarding the precision and reliability of parental recall of BW and GA is discrepant. Although some studies have shown maternal recall to be reliable,^10–13^ others suggest a bias, with poorer recall from mothers with more than one child or who are not of White European origin.^14,15^ This study provided a unique opportunity to examine the feasibility of collecting essential information relating to birth status from both parents and GPs using data collected from the Size and Lung function In Children (SLIC) study, which is the largest study of lung function undertaken in a multi-ethnic population of London primary schoolchildren to date.

The aims of this study were to (1) determine the feasibility of collecting BW and GA from parents and general practitioners (GP) in primary care surgeries, where all children are registered for health care; (2) assess the agreement of BW and GA data between GPs and parental recall; and (3) estimate the extent to which reliance on parental data may bias identification of full-term (i.e., ⩾37 weeks GA) and appropriately grown (i.e., ⩾2.5 kg BW) children for epidemiological studies, on the basis of data collected as part of the SLIC study.^16^

## Materials and Methods

As part of the SLIC study^16^ (www.ucl.ac.uk/slic), an epidemiological study designed to explore ethnic differences in lung function and body size in a multi-ethnic population of London children, anthropometry and spirometry were undertaken in primary schoolchildren between December 2010 and July 2013. Primary schools in the London area with a high ethnic mix of pupils were identified and ranked by education performance within boroughs. The sampling was undertaken from each stratum of rankings to ensure a wide range of socioeconomic circumstances. In this study, an all-inclusive strategy was adopted to ensure that no child would feel excluded from a study that was being undertaken in the school. Thus, all children with written parental consent (*n*=2,291) were eligible to participate in the study. The study was approved by the London-Hampstead Research Ethics Committee (REC: 10/H0720/53). Parents were requested to complete a study questionnaire that was sent home with the children. The information requested included relevant health information such as birth data and respiratory and medical history, ethnicity and socio-economic circumstances. A member of the study team was available to assist in person or over the phone in cases which required assistance in completing the questionnaire. Birth weight was reported in kilograms and grams or pounds and ounces (the latter being subsequently converted into kilograms and grams).^16^ Children were classified into four main ethnic groups—White (European descent), Black African origin (Black African or Black Caribbean descent), South Asian (Indian subcontinent) and Other/mixed ethnicities—on the basis of the child’s ethnicity information from parental questionnaires (PQs).

Socioeconomic circumstances (SEC) were assessed at the area level using the English Index of Multiple Deprivation (IMD)^17–19^ and at the individual level using the Family Affluence Scale (FAS).^19,20^ Each child was assigned an IMD using area postcodes for both their registered GP surgery and their home address to examine potential associations of any bias between GP or parental data according to locality. The FAS, commonly used for collecting socioeconomic data from children, included information such as the number of cars and computers owned by the family, whether the child shared a bedroom^17,19^ and the dominant language spoken within the household.

In cases for which parental consent was obtained for access to the child’s and maternal GP records, GP surgeries were requested either to extract the relevant birth data from medical records or to permit a designated researcher to extract such data. Approval from the relevant Primary Care Trusts was obtained to access GP records, with supplementary funding for service support costs being provided by the Local Comprehensive Research Networks to enable remuneration to be offered to GP surgeries.

### Statistical analysis

For the purposes of this study, GP data were used as baseline, with discrepancies between PQ and GP exceeding 0.10 kg for BW or 2 weeks for GA being considered to be of potential clinical or physiological significance.^10,11^ These thresholds were used to estimate the degree of potential underestimation and overestimation from the PQ, if the GP report was assumed to be correct. Children were also classified as being of LBW (<2.5 kg) and/or preterm (<37 weeks’ GA) according to both GPs and parental report. The Mann–Whitney *U*-test and binary logistic regression models were used to assess whether the child’s test age or socioeconomic circumstance distribution varied between GPs who (a) did or did not respond to requests for data and (b) could or could not provide the relevant birth data. An agreement between BW and GA reported by parents and GPs was assessed using the Bland and Altman method.^21^ Agreement between LBW and preterm classification according to PQ and GP was measured using the Kappa statistic. Multinomial logistic regression models were used to evaluate the extent and nature of any apparent parental misreport of birth information. Significance level was set at 0.05 and SPSS and R program were used for analyses.^22,23^ Data were stored in a dedicated research database (Re-Base software, Re-Base Ltd).

## Results

**Figure 1:**
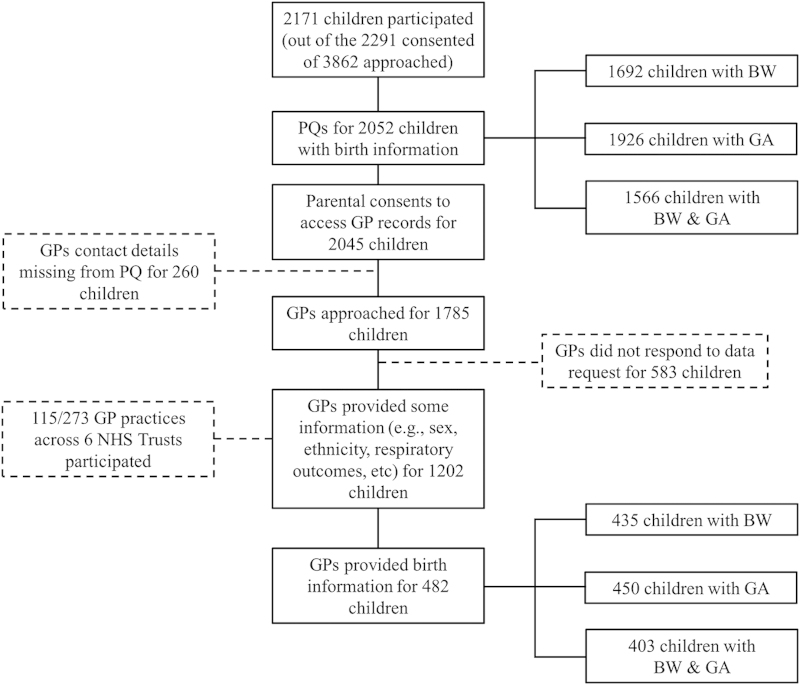
Study participation and birth data retrieval from parental recall and general practitioner. In all, 376 children had paired information (i.e., PQ and GP) for BW; 407 children had paired information for GA; and 322 children had paired information for BW and GA. BW, birth weight; GA, gestational age; GP, general practitioner; NHS, National Health Service; PQ, parental questionnaire.

Out of the 2,291 children with parental consent, 2,171 children (median age 8.1 (range 5.2–12.0) years; 47% boys) participated in the SLIC study.^16^ Of these, parental reports for BW and/or GA were available for 2,052 (95%) children, with 2,045 (94%) parents giving consent to access GP records. Of those with parental consent, 260 (13%) contact details for GPs were missing, and therefore GPs for 1,785 children were approached. Although some GP information regarding past medical history was available for 1,202 children (67% of requested), birth data were only available for 482 children (27% of those with parental consent and GP details, and 22% of the total study sample). Paired data (parent and GP) were available from 376 records for BW and 407 for GA, representing only 18 and 20% of those with parental consent to access GP data. Data availability from both sources is summarised in Figure 1.

The sex and age distributions were similar for children with or without GP data (median age (95% confidence interval (CI)): 8.1(8.0; 8.2) vs. 8.3 (8.1; 8.6) years, respectively). GP data were more likely to be missing for children of ethnicities other than White, who were not born in the UK, where English was not the dominant language at home or who lived in the most deprived areas or were in low FAS households (Table 1). The distribution of area-level or individual-level SEC characteristics in terms of data availability was similar for all indices (Supplementary Table S1).

Among the 40% of GPs who responded, details regarding BW or GA were more frequently missing for older children (8.2 (95% CI: 8.1; 8.4) vs. 7.8 (7.7; 8.0) years, *P*<0.0001) and those from low FAS households (mean (95% CI)% missing data: 78 (69; 87)% for low FAS vs. 42 (36; 48)% for high FAS, *P*<0.0001; Table 2). The proportion of missing data was independently associated with age, country of birth, dominant language, IMD and FAS, with the adjusted odds for not obtaining data being higher for older children and those not born in UK, without English as the dominant home language or who were living in more deprived areas. Low FAS was related to increased odds of missing GP birth data after accounting for the variables mentioned above (Table 2).

**Figure 2:**
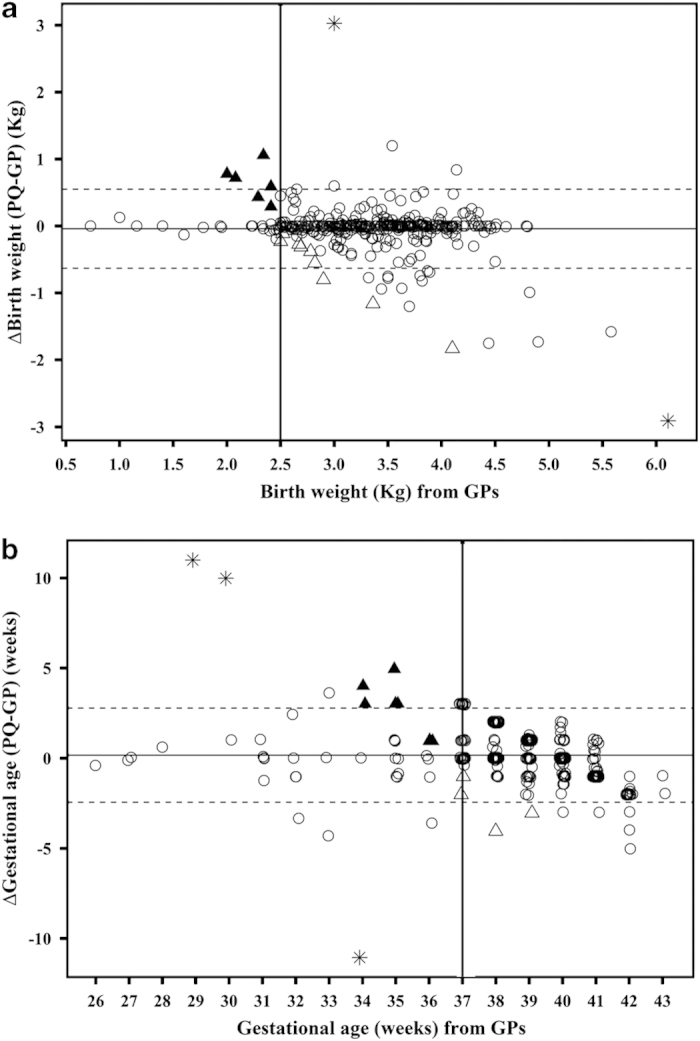
Difference in (**a**) birthweight and (**b**) gestational age between PQ and GP data versus GP data. For clarity, GP data were used as baseline and are thus plotted on the *x* axis, rather than the mean of GP and PQ data. Solid horizontal line represents the bias (i.e., mean difference) of the two methods, whereas dotted lines represent the 95% limits of agreement (LoA) between the two methods. Bold solid vertical lines indicate critical cutoffs of <2.5 kg and <37 weeks, which were used to categorise children having low birth weight or born preterm, respectively, according to GPs. ▲ symbols indicate children who would have been ‘misclassified’ as having normal birth weight (*n*=6) or born full term (*n*=8) if based on PQs rather than GP records. Δ symbols indicate children who would be potentially ‘misclassified’ as having low birth weight (*n*=11) or born preterm (*n*=4) if based on PQs rather than GP data. The outliers indicated by *, which obviously indicate misreporting by either PQ or GPs, have been excluded from the analyses. GP, general practitioner; PQ, parental questionnaire.

**Figure 3:**
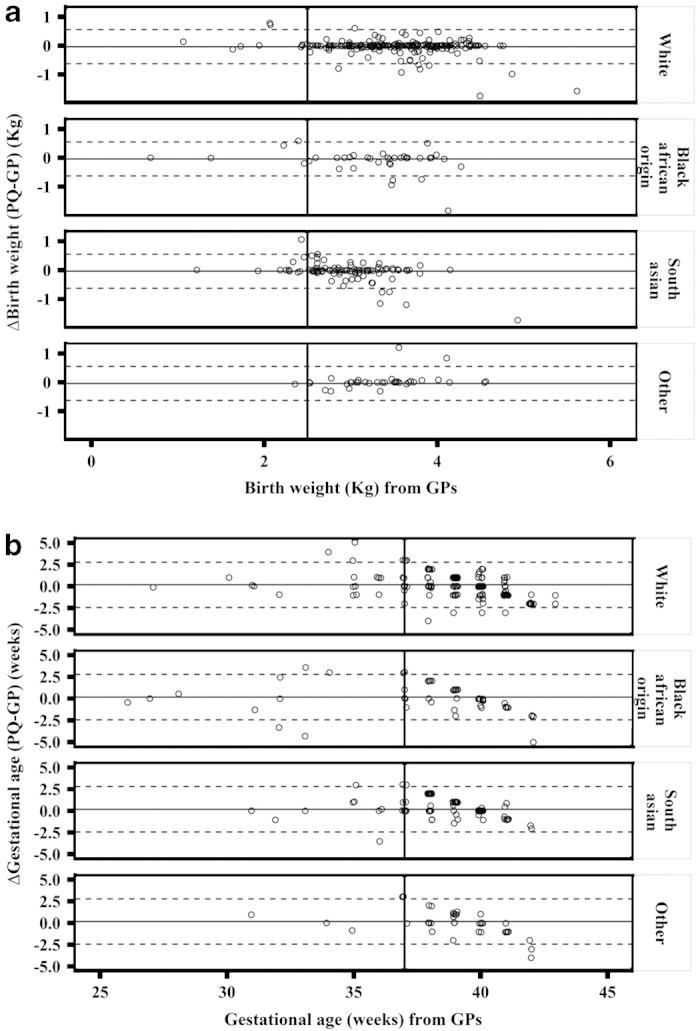
Differences between PQ and GP data with respect to (**a**) birth weight and (**b**) gestational age according to ethnicity. Solid horizontal lines represent the bias (i.e., mean difference) between the parental and GP data. Dashed lines represent the 95% limits of agreement between the two methods for the overall population. Bold solid vertical line indicates critical cutoffs of <2.5 kg for BW and <37 weeks for GA, which were used to categorise children who were having low birth weight or born preterm according to GPs. Points indicating extreme misclassification were excluded from this plot and analyses. GA, gestational age; GP, general practitioner; PQ, parental questionnaire.

There was no significant bias between PQ and GP reports of either birth weight (bias (95% CI): −0.04 (−0.07; −0.01) kg) or gestational age (0.17 (0.04; 0.30) weeks), but the relatively wide limits of agreement (95% LoA (95% CI): (−0.63 (−0.68; −0.58); 0.55 (0.50; 0.60) kg) for birth weight; (−2.4 (−2.6; −2.2); 2.8 (2.5; 3.0) weeks) for gestational age) indicate that individual differences may exist (Figure 2). Although there was a trend for parents to underestimate BW or GA for heavier and full-term children when compared with GP data (−0.4⩽*r*⩽−0.2, *P*⩽0.1 for all cases), the agreement regarding BW or GA was consistent across ethnicities, indicating that no ethnic bias was observed when estimating either BW or GA from PQ as compared with GP data (Figure 3). Differences in BW or GA were also found to be constant across the age range, although they were somewhat larger for children of LBW as compared with those of normal BW. Nevertheless, no trend towards over- or under-reporting of data from PQ was evident (rho<0.2, *P*>0.1 for both BW and GA). When the analysis was repeated after including the five extreme data points, results remained very similar albeit the limits of agreement were slightly wider.

Parental ‘underestimation’ of BW by at least 0.1 kg occurred in 19% (95% CI: 15; 23%) of children, whereas ‘overestimation’ occurred in 12% (9; 16%). By contrast, parents under- or over-reported GA by at least 2 weeks in 4% (3; 7%) and 3% (1; 4%) of children, respectively. The odds of parents underestimating BW were ~2.5 (95% CI: 1.1; 5.5) times higher in Black African-origin children when compared with White children. In addition, lower FAS and increasing IMD quintile were both associated with increased misclassification of birth weight status (Table 3). No significant associations were observed between socioeconomic circumstances and the likelihood of parents mis-estimating GA (Supplementary Table S2).

Nine percent (95% CI: 8; 11%) of children were classified as LBW and 6% (5; 8%) were classified as preterm by parents compared with 6% (4; 9%) and 9% (7; 12%), respectively, when classified by GPs. Among children with paired data, there was good agreement with respect to whether or not the child was of LBW (95.5%) or born preterm (97%) (Supplementary Table S3). When repeating the analysis including the five extreme data points, no difference was seen in the proportion of misclassification, and the agreement remained in the same level as the initial analysis. Significant association was found between parental mis-estimation of birth weight and socioeconomic circumstances as indicated by IMD and FAS, with those from most deprived areas or lower FAS having higher odds to mis-estimate the child’s birthweight. No significant associations were found with GA, and both of these results are in line with original data in the main manuscript.

## Discussion

### Main findings

These results demonstrate that it is currently not feasible to obtain essential birth data from GP records. Parental recall is an appropriate alternative, especially for birth weight. Comparison of GP data with PQ showed reasonable agreement on average. Although birth data from reliable health records would be undeniably preferable, our findings suggest that parental reports have the potential of yielding high-quality data and quicker access to the data, for both gestational age and birth weight.

### Feasibility of data retrieval

In contrast to the relative ease with which parental data were collected, obtaining birth data from GPs was difficult and information was less likely to be available if the child was older, born outside the UK or where English was not the dominant language at home. This raises a number of issues. First, owing to the overall low response from GPs, currently, this does not appear to be a feasible method for obtaining data for epidemiological studies. Further, the response rate was especially low for children from more deprived areas, thereby risking collection bias towards those with higher SEC. Second, the lower GP response rate for children not born in the UK or without English as their dominant home language may have an impact on the provision of health care for migrant children. Increasing awareness of the potential long-term influence of early-life events, including preterm birth and intrauterine growth restriction, highlights the need for GPs to try to obtain this information as accurately as possibly at the time of registration. Reassuringly, the barriers to obtaining information from the GP did not arise from parents. Not only was it feasible to collect birth data from virtually all parents via the questionnaire, but the vast majority gave consent for access to GP records, regardless of ethnicity or socioeconomic circumstance.

### Interpretation of findings in relation to previously published work

Comparison of paired PQ and GP data, where available, showed good agreement on average across all ethnic groups and socioeconomic circumstances. Previous studies found contrasting results. In the Millennium Cohort Study, although there was 94% agreement in the reporting of GA within 1 week between parent and medical record, disagreement was associated with low SEC.^24^ Similarly, significant underestimation of GA and less accurate BW reporting was found from mothers of non-white children.^11^ However, in both these studies and the current study, disagreement only resulted in minimal misclassification of birth status, suggesting that parental reporting of BW and GA is accurate enough to provide appropriate classification of birth status, especially within the context of large-scale epidemiological studies. It should be noted that although GP data were used as the baseline for the purpose of analysis, with parental ‘underestimation’ or ‘overestimation’ based on the assumption that GP data should be the most accurate, this assumption was not necessarily correct, as shown by several physiologically impossible birth data or ‘outliers’ provided by GPs (see Figure 2).

### Strengths and limitations of this study

The major limitation of this study was the relatively low sample size in certain sub-categories, mainly owing to the high proportion of GP missing data for children from more deprived households, meaning that potentially important differences pertaining to deprivation could not be discounted. In addition, although the best approach for collecting birth data would be via hospital birth records, this would only have been feasible for children born in England. Given the nature of the multi-ethnic SLIC study, many of the children had been born outside London or indeed outside the UK, thereby precluding this approach. The use of GP data was as a baseline reference for comparisons with PQ, rather than a gold standard method. Nevertheless, the SLIC study is the largest study of lung function undertaken in a multi-ethnic population of London primary schoolchildren to date, providing a unique opportunity to examine the feasibility of collecting essential information relating to birth status from both parents and GPs.

### Implications for future research, policy and practice

A major current focus of the National Health Service in the UK is the development of administrative and informatics networks to develop linked electronic health records for both clinical (http://www.england.nhs.uk/ourwork/tsd/sst/) and public health research (http://www.cprd.com/intro.asp) purposes. The intention is that data held in electronic health records be available to appropriate individuals via remote access, thus facilitating record/data access. However, contrary to time projections at the inception of the SLIC study, the software enabling this functionality has yet to be rolled out to the majority of GP surgeries in London. It was therefore impossible to access health records without the cooperation of the GP surgeries. Unfortunately, among the GPs contacted, ~1/3 failed to respond at all to requests for children’s birth data. This was despite providing written parental consent for access to specific medical records, monetary incentives for participation and the option for a researcher to extract the necessary information. The reason for such a high rate of non-response is unclear, but it may reflect either lack of resources or failure to appreciate the relevance of requests for such data. Inner-city surgeries in deprived areas may find it difficult to engage with activities perceived to be research-based, and the quality of data collected by GP practice nurses may be compromised both by competing clinical priorities or lack of research-active practitioners.^25^ There is a shift to electronic health records in the UK; however, even when fully functional, the issue of missing data for those not born in the UK will still remain.

### Conclusions

Detailed BW and GA data were difficult to retrieve from GP records. The proportion of missing birth data from GP records emphasises the need for more accurate and systematic recording of these data. Furthermore, it is essential that electronic health records be established within health care systems and that the information be made readily available through the primary care practitioners to the appropriate personnel. Parental report of birth data, at least for primary schoolchildren, is an appropriate alternative to health records for use in obtaining high-quality data for epidemiological studies.

## Figures and Tables

**Table 1:** Factors associated with GPs non-response upon request for information

	*Number of GPs contacted* a	*Non-response (%)*	*Univariable models*	
			*OR (95% CI)*	P^b^
*Sex*				
Girls	954	34	Baseline	
Boys	831	32	0.92 (0.75; 1.12)	0.40
				
*Ethnicity*				
White	637	20	Baseline	
Black African origin	458	38	2.49 (1.89; 3.26)	<0.0001
South Asian	474	43	3.12 (2.39; 4.07)	<0.0001
Other	216	36	2.25 (1.60; 3.16)	<0.0001
				
*Born in UK*				
Yes	1,514	32	Baseline	
No	243	41	1.52 (1.15; 2.01)	0.003
				
*Dominant language in the family*				
English	828	28	Baseline	
Other	360	37	1.56 (1.20; 2.03)	0.001
				
*Family’s IMD domain*^c^				
1st quintile (least deprived)	90	12	Baseline	
2nd quintile	258	32	3.35 (1.69; 6.63)	<0.0001
3rd quintile	253	34	3.70 (1.87; 7.32)	<0.0001
4th quintile	548	38	4.33 (2.25; 8.32)	<0.0001
5th quintile (most deprived)	632	31	3.23 (1.68; 6.20)	<0.0001
				
*FAS*^c^^,^^d^				
High (5–6)	393	27	Baseline	
Moderate (2–4)	1,135	33	1.34 (1.04; 1.72)	0.03
Low (0–1)	137	39	1.74 (1.16; 2.62)	0.008

Abbreviations: FAS, family affluent score; GP, general practitioner; IMD, index of multiple deprivation; OR (95% CI), odds ratio (95% confidence interval).

a No feedback was received from GPs for 583/1,785 (33%) children for whom there was parental consent to access records (see Figure 1).

b *P* values derived through univariable logistic regression models to evaluate the factors related with the likelihood of non-response.

c Detailed information regarding the IMD distribution of income and GP domain and the individual components for FAS is presented in Supplementary Table S1.

d FAS was grouped in three categories owing to the small sample size in the lower scores.

**Table 2:** Factors associated with missing birth data from GP records received

	*Number of GPs who responded* a	*Missing birth data (%)*	*Univariable models*		*Multivariable model*	
			*OR (95% CI)*	P^b^	*OR (95% CI)*	P^b^
Child’s age (per year)			1.20 (1.12; 1.30)	<0.0001	1.14 (1.04; 1.26)	0.007
						
*Sex*						
Girls	634	61	Baseline		Baseline	
Boys	568	58	0.89 (0.71; 1.12)	0.33	1.12 (0.82; 1.53)	0.49
						
*Ethnicity*						
White	511	53	Baseline		Baseline	
Black African origin	284	75	2.61 (1.90; 3.59)	<0.0001	1.55 (0.95; 2.53)	0.08
South Asian	268	55	1.08 (0.80; 1.45)	0.63	0.50 (0.33; 0.77)	0.002
Other	139	65	1.63 (1.10; 2.40)	0.01	1.60 (0.97; 2.64)	0.07
						
*Born in UK*						
Yes	1,037	56	Baseline		Baseline	
No	143	86	4.88 (3.00; 7.96)	<0.0001	5.00 (2.59; 9.65)	<0.0001
						
*Dominant language in family*						
English	600	50	Baseline		Baseline	
Other	226	72	2.58 (1.86; 3.59)	<0.0001	1.71 (1.12; 2.62)	0.01
						
*Family’s IMD domain* c						
1st quintile (least deprived)	79	39	Baseline		Baseline	
2nd quintile	176	39	0.98 (0.57; 1.68)	0.93	1.10 (0.57; 2.10)	0.78
3rd quintile	167	41	1.09 (0.63; 1.88)	0.76	0.86 (0.44; 1.67)	0.65
4th quintile	342	68	3.31 (2.00; 5.49)	<0.0001	2.51 (1.31; 4.80)	0.006
5th quintile (most deprived)	436	73	4.17 (2.54; 6.87)	<0.0001	2.17 (1.15; 4.12)	0.02
						
*FAS* c d						
High (5–6)	286	42	Baseline		Baseline	
Moderate (2–4)	757	64	2.43 (1.84; 3.20)	<0.0001	1.59 (1.10; 2.30)	0.01
Low (0–1)	83	78	5.00 (2.82; 8.86)	<0.0001	2.86 (1.27; 6.43)	0.01

Abbreviations: FAS, family affluent score; GP, general practitioner; IMD, index of multiple deprivation; OR (95% CI), odds ratio (95% confidence interval).

a GPs who responded could not provide any data on BW or GA for 720/1,202 (60%) children (see Figure 1).

b *P* values derived through univariable or multivariable logistic regression models to evaluate the factors related with the likelihood of missing birth data. Multivariable model was adjusted for age, sex, ethnicity, country of birth, language, family’s IMD domain and FAS.

c Detailed information regarding the IMD distribution of income and GPs domain and the individual components for FAS is presented in Supplementary Table S1.

d FAS was grouped in three categories owing to the small sample size in the lower scores.

**Table 3:** Factors associated with the likelihood of parental ‘misclassification’^a^ of child’s birth weight

*Univariable multinomial regression models* b	*Underestimation (*n*=70)*^c^		*Overestimation (*n*=45)*^c^	
	*OR (95% CI)*	P	*OR (95% CI)*	P
Child’s age (per year)	1.14 (0.97; 1.34)	0.13	1.17 (0.96; 1.42)	0.13
				
*Sex (baseline: girls)*				
Boys	1.23 (0.73; 2.10)	0.44	1.09 (0.58; 2.05)	0.80
				
*Ethnicity (baseline: white)*				
Black African origin	2.48 (1.12; 5.51)	0.03	1.55 (0.53; 4.51)	0.42
South Asian	1.57 (0.84; 2.92)	0.16	1.57 (0.75; 3.26)	0.23
Other	0.65 (0.21; 1.98)	0.45	0.97 (0.31; 3.04)	0.96
				
*Born in UK (baseline: yes)*				
No	0.82 (0.17; 3.87)	0.80	1.29 (0.27; 6.19)	0.75
				
*Dominant language in family (baseline: English)*				
Other	1.42 (0.64; 3.13)	0.38	0.82 (0.27; 2.51)	0.73
				
*Family’s IMD domain (baseline: 1st & 2nd quintile (least deprived))* d				
3rd quintile	1.76 (0.80; 3.88)	0.16	0.94 (0.38; 2.33)	0.90
4th quintile	3.24 (1.51; 6.92)	0.002	1.76 (0.76; 4.08)	0.19
5th quintile (most deprived)	4.22 (2.00; 8.91)	<0.0001	1.80 (0.75; 4.28)	0.19
				
*FAS (baseline: high 5–6)*^e^				
Moderate (2–4)	1.66 (0.92; 3.00)	0.10	1.17 (0.60; 2.3)	0.65
Low (0–1)	11 (1.9; 65)	0.007	13 (2.2; 77)	0.004

Abbreviations: CI, confidence interval; FAS, family affluent score; GP, general practitioner; IMD, index of multiple deprivation; OR, odds ratio.

a Parental ‘misclassification’ was defined as a difference in child’s BW of more than 0.10 kg compared with the GP records. For the purpose of this analysis, it was presumed that GP data would be the more accurate, but as mentioned in the discussion this assumption was not necessarily always correct.

b Modelling was based on 376 cases for which paired data were available.

c The middle category (i.e., those neither underestimated nor overestimated by PQ) was used as the baseline against which the other two were compared.

d The 1st and 2nd quintile of IMD were grouped together owing to the small sample size in the 1st quintile.

e FAS was grouped in three categories owing to the small sample size in the lower scores.

## Notes

SH reports personal fees as an editor of the Journal of Ethnicity and Health, outside the submitted work. CG is an Assistant Editor of *npj Primary Care Respiratory Medicine*, but was not involved in the editorial review of, nor the decision to publish, this article. The remaining authors declare no conflict of interest.
