# Supplementary material for: Birth data accessibility via primary care health records to classify health status in a multi-ethnic population of children: an observational study
Source: NPJ Prim Care Respir Med. 2015 Jan 22;25:14112–. doi: 10.1038/npjpcrm.2014.112 (PMC4353844; doi:10.1038/npjpcrm.2014.112)
Supplement: Supplementary Tables [file npjpcrm2014112-s1.doc]

# Birth data accessibility via primary care health records to classify health status in a multi-ethnic population of children: an observational study

# ^1^Bonner, Rachel*; ^1^Bountziouka, Vassiliki*; ^1^Stocks, Janet; ^2^Harding, Seeromanie; ^3^Wade, Angela; ^4^Griffiths, Chris; ^1,5^Sears, David; ^1,6^Fothergill, Helen; ^1,7^Slevin, Hannah; ^1^Lum, Sooky

*Joint first authors

^1^Respiratory, Critical Care & Anaesthesia Section (Portex Unit), UCL, Institute of Child Health, London, UK

^2^Clinical Epidemiology, Nutrition and Biostatistics Section, UCL, Institute of Child Health, London, UK

^3^MRC/CSO, Social and Public Health Sciences Unit, University of Glasgow

^4^Asthma UK Centre for Applied Research, Centre for Primary Care and Public Health, Queen Mary's School of Medicine and Dentistry, University of London, UK

^5^Lung Function Unit, Royal Brompton Hospital, London, UK

^6^Torbay Hospital, South Devon NHS Trust, Devon, UK

^7^ Faculty of Medicine, University of Southampton, Southampton, UK

**Supplementary Information**

**Table S1:** Distribution of socio-economic circumstances according to GPs response to request for information.

|  | Total GPs contacted | **Missing GP responses^┬^** | P | Total GPs respond | **Missing GP birth data^┴^** | P |
| --- | --- | --- | --- | --- | --- | --- |
|  | N | % (95% CI) |  | N | % (95% CI) |  |
| **GP's IMD domain** |  |  | <0.0001 |  |  | <0.0001 |
| *1st quintile (least deprived)* | 23 | 26 (8; 44) |  | 17 | 18 (0; 36) |  |
| *2nd quintile* | 397 | 25 (21; 29) |  | 298 | 41 (35; 46) |  |
| *3rd quintile* | 143 | 47 (39; 55) |  | 76 | 58 (47; 69) |  |
| *4th quintile* | 511 | 41 (36; 45) |  | 304 | 57 (51; 62) |  |
| *5th quintile (most deprived)* | 663 | 31 (27; 34) |  | 459 | 73 (69; 77) |  |
| **Family’s IMD income** |  |  | <0.0001 |  |  | <0.0001 |
| *1st quintile (least deprived)* | 83 | 11 (4; 18) |  | 74 | 34 (23; 45) |  |
| *2nd quintile* | 169 | 37 (29; 44) |  | 107 | 43 (34; 52) |  |
| *3rd quintile* | 204 | 28 (22; 35) |  | 146 | 38 (30; 46) |  |
| *4th quintile* | 578 | 35 (31; 39) |  | 374 | 62 (57; 67) |  |
| *5th quintile (most deprived)* | 747 | 33 (30; 37) |  | 499 | 73 (69; 76) |  |
| **Number of computers (incl. laptops etc)/ household** |  |  | 0.29 |  |  | <0.0001 |
| *> 2* | 435 | 33 (29; 38) |  | 291 | 49 (44; 55) |  |
| *2* | 592 | 33 (29; 36) |  | 399 | 58 (54; 63) |  |
| *1* | 626 | 31 (28; 35) |  | 430 | 67 (62; 71) |  |
| *0* | 27 | 41 (22; 59) |  | 16 | 69 (46; 91) |  |
| **Number of vehicles/ household** |  |  | <0.0001 |  |  | <0.0001 |
| *≥ 2* | 416 | 29 (25; 33) |  | 295 | 43 (37; 48) |  |
| *1* | 882 | 33 (30; 36) |  | 588 | 60 (56; 64) |  |
| *0* | 385 | 34 (29; 38) |  | 256 | 77 (71; 82) |  |
| **Child's own bedroom** |  |  | 0.02 |  |  | <0.0001 |
| *Yes* | 911 | 30 (27; 33) |  | 639 | 55 (51; 59) |  |
| *No* | 776 | 35 (32; 38) |  | 505 | 66 (62; 70) |  |

^┬^No feedback was received from GPs for 583/1785 (33%) children for whom there was parental consent to access records.

^┴^ GPs who did respond could not provide any data on BW or GA for 720/1202 (60%) children (see *Figure 1*). P-values derived through chi-square test to evaluate factors associated with a) GP’s non-response and b) lack of birth data.

Table S2: Factors associated with the likelihood of parental misclassification^∫^ of child’s gestational age.

| Univariable multinomial regression models^├^ | Underestimation (n=18)^┼^ | | Overestimation (n=10)^┼^ | |
| --- | --- | --- | --- | --- |
|  | OR (95% CI) | P | OR (95% CI) | P |
| Child’s Age (per year) | 1.09 (0.73; 1.63) | 0.68 | 1.06 (0.79; 1.44) | 0.69 |
| Sex (baseline: girls) |  |  |  |  |
| *Boys* | 1.08 (0.31; 3.80) | 0.90 | 0.87 (0.33; 2.24) | 0.77 |
| Ethnicity (baseline: white) |  |  |  |  |
| *Black-African origin* | 2.68 (0.58; 12) | 0.21 | 2.55 (0.78; 8.35) | 0.12 |
| *South Asian* | 0.47 (0.05; 4.24) | 0.50 | 0.80 (0.20; 3.17) | 0.75 |
| *Other* | 2.63 (0.46; 15) | 0.28 | 2.25 (0.56; 9.11) | 0.26 |
| Born in UK (baseline: Yes) |  |  |  |  |
| *No* | 2.06 (0.25; 17) | 0.72 | 1.42 (0.18; 11) | 0.74 |
| Dominant language in family (baseline: English) |  |  |  |  |
| *Other* | 0.61 (0.07; 4.95) | 0.64 | 1.64 (0.43; 6.28) | 0.47 |
| Family’s IMD domain (baseline: 1st & 2nd quintile (least deprived))^┴^ |  |  |  |  |
| *3rd quintile* | 1.04 (0.17; 6.37) | 0.97 | 0.26 (0.03; 2.20) | 0.22 |
| *4th quintile* | 0.35 (0.04; 3.15) | 0.35 | 0.93 (0.25; 3.38) | 0.91 |
| *5th quintile (most deprived)* | 2.52 (0.58; 11) | 0.22 | 1.76 (0.57; 5.41) | 0.33 |
| FAS (baseline: High 5-6)^┬^ |  |  |  |  |
| *Moderate (2-4)* | 1.09 (0.26; 4.64) | 0.91 | 1.64 (0.50; 5.32) | 0.41 |
| *Low (0-1)* | 2.32 (0.24; 22) | 0.84 | 4.93 (0.83; 29) | 0.08 |

Abbreviations: OR: Odds ratio, GP: General Practitioner; IMD = Index of multiple deprivation; FAS = Family affluent score.

^∫^ For the purpose of this analysis, parental misclassification was defined as a difference in child’s GA of more than 2 weeks compared with the GP records. As discussed in the main MS, this presumption was not necessarily always correct, and it is possible that in the presence of transcription errors by the GP, the degree of parental ‘misclassification’ was even smaller than presented here. ^├^Modelling was based on 407 cases for which paired data were available. ^┼^The middle category (i.e. those neither under nor over estimated by PQ) was used as the baseline against which the other two were compared.

^┴^The 1^st^ and 2^nd^ quintile of IMD were grouped together due to the small sample size in the 1^st^ quintile. ^┬^FAS was grouped in three categories due to the small sample size in the lower scores.

Table S3: Classification of children’s birth status using parental questionnaire or general practitioner’s records.

Paired information regarding:

1. Birthweight (n=376)

|  | GP | | |
| --- | --- | --- | --- |
|  |  | LBW | Normal BW |
| PQ | LBW | 4.8% (18/376) | 2.9% (11/376) |
|  | Normal | 1.6% (6/376) | 90.7% (341/376) |

Kappa (95% CI)=0.66 (0.50; 0.81), p<0.001

1. Gestational Age (n=407)

|  |  | GP | |
| --- | --- | --- | --- |
|  |  | Preterm | Fullterm |
| PQ | Preterm | 7.6% (31/407) | 1.0% (4/407) |
|  | Fullterm | 2.0% (8/407) | 89.4% (364/407) |

Kappa=0.82 (0.72; 0.92), p<0.001

1. Both sources (n=322)

|  | **Birth info classification from GP** | | | Total |
| --- | --- | --- | --- | --- |
|  | Normal BW & GA | low BW or preterm | low BW & preterm |  |
| **Birth info classification from PQ** |  |  |  |  |
| Normal BW & GA | 282 | 9 | 0 | 291 |
| low BW or preterm | 10 | 7 | 1 | 18 |
| low BW & preterm | 1 | 2 | 10 | 13 |
| Total | 293 | 18 | 11 | 322 |

Kappa=0.59 (0.44; 0.74), p<0.001

**Abbreviations:** BW: Birth weight, GA: Gestational age, PQ: Parental questionnaire, GP: General Practitioner of primary care, LBW: low birthweight.

Critical cut-offs of <2.5 kg and <37 weeks were used to categorise children born low birthweight or preterm respectively. Of the 376 children with paired information on BW 1.6% children were misclassified as normal BW according to PQ records while classified as LBW using GPs and 2.9% were misclassified as LBW using the PQ records while classified as normal BW by GPs (Table S3a)). Of the 407 children with paired information on GA, 2.0% were misclassified as normal GA using the PQ records while classified as preterm using GP data and 1.0% were misclassified as preterm using the PQ records while classified as normal GA using GP data (Table S3b)). On further data exploration, based on the paired birth data available, 9/291 (3%) would have been classified as normal BW by PQ but of LBW/preterm by the GP, while 11/293 (3.7%) were classified as LBW or preterm by PQ while classified as normal by GP. Due to this low mis-classification rate we still feel that parental recall is an appropriate alternative (Table S3c)).
